# Periplasmic Expression of 4/7 α-Conotoxin TxIA Analogs in *E. coli* Favors Ribbon Isomer Formation – Suggestion of a Binding Mode at the α7 nAChR

**DOI:** 10.3389/fphar.2019.00577

**Published:** 2019-05-31

**Authors:** Yamina El Hamdaoui, Xiaosa Wu, Richard J. Clark, Julien Giribaldi, Raveendra Anangi, David J. Craik, Glenn F. King, Sebastien Dutertre, Quentin Kaas, Volker Herzig, Annette Nicke

**Affiliations:** ^1^Walther Straub Institute of Pharmacology and Toxicology, Faculty of Medicine, Ludwig-Maximilians-Universität München, Munich, Germany; ^2^Institute for Molecular Bioscience, The University of Queensland, Brisbane, QLD, Australia; ^3^School of Biomedical Sciences, The University of Queensland, Brisbane, QLD, Australia; ^4^CNRS, Institut des Biomolécules Max Mousseron, UMR 5247, Université de Montpellier, Montpellier, France

**Keywords:** *E. coli*, recombinant expression, 4/7 α-conotoxin, ribbon isomer, molecular modeling, NMR spectroscopy

## Abstract

Peptides derived from animal venoms provide important research tools for biochemical and pharmacological characterization of receptors, ion channels, and transporters. Some venom peptides have been developed into drugs (such as the synthetic ω-conotoxin MVIIA, ziconotide) and several are currently undergoing clinical trials for various clinical indications. Challenges in the development of peptides include their usually limited supply from natural sources, cost-intensive chemical synthesis, and potentially complicated stereoselective disulfide-bond formation in the case of disulfide-rich peptides. In particular, if extended structure–function analysis is performed or incorporation of stable isotopes for NMR studies is required, the comparatively low yields and high costs of synthesized peptides might constitute a limiting factor. Here we investigated the expression of the 4/7 α-conotoxin TxIA, a potent blocker at α3β2 and α7 nicotinic acetylcholine receptors (nAChRs), and three analogs in the form of maltose binding protein fusion proteins in *Escherichia coli*. Upon purification via nickel affinity chromatography and release of the toxins by protease cleavage, HPLC analysis revealed one major peak with the correct mass for all peptides. The final yield was 1–2 mg of recombinant peptide per liter of bacterial culture. Two-electrode voltage clamp analysis on oocyte-expressed nAChR subtypes demonstrated the functionality of these peptides but also revealed a 30 to 100-fold potency decrease of expressed TxIA compared to chemically synthesized TxIA. NMR spectroscopy analysis of TxIA and two of its analogs confirmed that the decreased activity was due to an alternative disulfide linkage rather than the missing C-terminal amidation, a post-translational modification that is common in α-conotoxins. All peptides preferentially formed in the ribbon conformation rather than the native globular conformation. Interestingly, in the case of the α7 nAChR, but not the α3β2 subtype, the loss of potency could be rescued by an R5D substitution. In conclusion, we demonstrate efficient expression of functional but alternatively folded ribbon TxIA variants in *E. coli* and provide the first structure–function analysis for a ribbon 4/7-α-conotoxin at α7 and α3β2 nAChRs. Computational analysis based on these data provide evidence for a ribbon α-conotoxin binding mode that might be exploited to design ligands with optimized selectivity.

## Introduction

Nicotinic acetylcholine receptors (nAChRs) are members of the Cys-loop superfamily of pentameric ligand-gated ion channels. Whereas the muscle-type receptors consist of four different subunits (α1,β1,γ/ε,δ), the assembly and stoichiometry of the 11 cloned neuronal subunits (α2–α7, α9, α10, and β2–β4) into nAChR subtypes is considerably more diverse and not well-defined. These neuronal nAChRs represent important drug targets such as for the treatment of pain, Alzheimer’s disease, and nicotine addiction (Dineley et al., 2015; Lombardo and Maskos, 2015; Mohamed et al., 2015; Giribaldi and Dutertre, 2018; Hone and McIntosh, 2018). Deciphering their specific compositions and location in nervous system tissues as well as their respective physiological roles requires highly selective ligands.

Venoms from animals contain complex mixtures of small proteins and peptides that are often structurally constrained by disulfide bridges. Some of these peptides show high potency and specificity for certain ion channel subtypes. Cone snails are predatory marine animals that have evolved numerous peptide families that target various ligand- and voltage-gated ion channels (Akondi et al., 2014). The best-studied conotoxin family from a pharmacological perspective is the α-conotoxins, members of which are competitive antagonists of nAChRs (Dutertre et al., 2017). Some of these peptides have analgesic activity *in vivo* and thus are important lead structures for drug development (Akondi et al., 2014; Mohammadi and Christie, 2015).

The majority of α-conotoxins are composed of 12–19 amino acid residues including four cysteine residues that form two disulfide bonds. The cysteines are arranged in a CC–C–C pattern that defines the conotoxin Cysteine Framework I (Kaas et al., 2010). This framework is characterized by vicinal Cys1 and Cys2 residues and two loops formed by Cys1–Cys3 and Cys2–Cys4 disulfide bridges (referred to as the “globular” conformation). Based on the number of amino acid residues within the two loops, the currently characterized α-conotoxins are further classified into 3/4, 4/4, 3/5, 4/6, and 4/7 α-conotoxin subfamilies. These subfamilies show some common specificity for certain nAChR subtypes, with for example, the 3/5 α-conotoxins targeting the muscle-type nAChR and most identified 4/7 α-conotoxins preferentially targeting α7 and/or α3β2^∗^ neuronal nAChRs (^∗^ indicates the potential presence of further subunits) (Dutertre et al., 2017). Understanding the structure-activity relationships of conotoxins might aid in the development of optimized peptides with tailored selectivity. Usually, such studies employ chemical synthesis for the production of modified versions of the toxins. However, the production of multiple analogs or large quantities for automated application systems or preclinical treatment studies is costly, as is the production of large quantities of isotopically enriched samples for high resolution NMR spectroscopy studies or metabolic flux analysis (Antoniewicz, 2015). Chemical synthesis is also tedious if done manually and requires special equipment and experience that is not typically found in molecular biology laboratories. More generally, in the case of larger peptides (>40 aa), the yield from chemical synthesis is typically low. Finally, certain native peptides are inherently difficult to produce synthetically.

Venom-peptide production in heterologous expression systems might provide an efficient and economical alternative to chemical synthesis for molecular biology laboratories (Klint et al., 2013). It might also be suitable for large scale commercial toxin production. In the current study, we adapted an *Escherichia coli* periplasmic expression system (Klint et al., 2013) for the production of 4/7 α-conotoxin TxIA and three analogs. Unexpectedly, the functional and structural characterization of the expressed analogs indicated that they adopt a fold different from the native peptide (i.e., a 1–4, 2–3 “ribbon” rather than a 1–3, 2–4 “globular” disulfide conformation). These data demonstrate the need for careful structural analysis and confirm earlier findings that non-native folds could still be active. As an explanation for this activity and a basis for future structural studies, we provide the first NMR solution structure of a 4/7 α-conotoxin ribbon isomer and propose a binding mode for this peptide at the α7 nAChR. This information might be useful for the design of new lead structures based on the ribbon α-conotoxin scaffold.

## Materials and Methods

### Preparation of Plasmids

A nucleotide sequence encoding a tobacco etch virus (TEV) protease recognition site (ENLYFQG) followed by [R5N,I9H] TxIA and including *KpnI* and *SacI* restrictions sites at the 5′ and 3′ ends, respectively, was optimized for expression in *E. coli* and assembled from synthetic oligonucleotides (Life Technologies). This construct was cloned in-frame to an 5′ His_6_-maltose binding protein (MBP) sequence into the pLICC_D168 vector (Anangi et al., 2012) using *KpnI* and *SacI* cloning sites. The conserved N-terminal glycine residue of the conotoxin sequence forms the last amino acid of the TEV protease recognition site and thus, upon cleavage, produces a native N-terminus in the conotoxin. Plasmids for production of TxIA as well as single mutated R5D and R5N analogs were obtained from this construct by site-directed mutagenesis. The sequences of all plasmids were confirmed by Sanger sequencing.

### Recombinant Expression of TxIA Analogs in *E. coli*

α-Conotoxin expression in *E. coli* was performed using a previously described method (Anangi et al., 2012; Klint et al., 2013). BL21 strains of *E. coli* were transformed with the respective plasmids using a standard heat shock protocol. Five colonies were selected to inoculate 5 ml culture medium (Luria-Bertani plus ampicillin) and grown at 37°C until the optical density (OD_600_) reached 1.0. To determine optimal conditions for toxin expression, 4 ml × 1 ml samples of each culture were then diluted and grown again in 5 ml cultures until an OD of 0.6–0.8 was reached. Then heterologous protein expression was induced with IPTG. In doing so, culturing at 16°C and 37°C and induction with 0.5 and 1.0 mM IPTG were performed and protein expression in induced and non-induced cultures was compared after 4 or 12 h by SDS-PAGE analysis of a cell pellet from 500 μl of culture medium. The clone with the highest protein expression was selected and used to start 1–2 l cultures (from 50 to 100 ml precultures), which were then grown at 37°C with shaking at ∼220 rpm. After reaching an OD of 0.6–0.8, toxin expression was induced with 1 mM IPTG and cultures were grown overnight at 16°C. Cultures were then centrifuged (15 min at 10,500 *g*) and bacterial pellets frozen at −80°C.

### SDS-PAGE Analysis

500 μl of culture was centrifuged [5 min, 3,381 *g* (6,000 rpm) in a desktop centrifuge] and cell pellets were supplemented with 30 μl SDS running buffer and 30 μl 3× loading buffer. After heating (10 min at 95–100°C), 30 μl of the solubilized cells were separated on a 10% SDS-PAGE gel under reducing conditions (5% β-mercaptoethanol in the sample). To control the purification and cleavage process, equivalent volumes (30–200 μl) of lysate, flow-through, eluate, and cleaved sample were supplemented with SDS running buffer to obtain 200 μl. After addition of 200 μl 3× SDS running buffer, 50 μl of each sample were separated using SDS-PAGE.

### Recombinant Peptide Purification and Cleavage

Cell pellets were resuspended in 50 ml equilibration buffer (TN: 25 mM Tris-HCl, 300 mM NaCl, pH 7.0) and lysed in a constant-pressure cell disrupter (27 kpsi, TS Series Cell Disrupter, ConstantSystems, Ltd., Daventry, United Kingdom). After centrifugation (4°C, 30 min, 44,267 *g*), the supernatant was diluted with an equal volume of modified TN buffer (40 mM Tris-HCl, 400 mM NaCl, pH 8.0), supplemented with DNAse (1 μg/ml lysate) and incubated for 30 min with 5 ml Ni-NTA Superflow resin (Qiagen Pty, Ltd., Chadstone, VIC, Australia) in a gravity-fed column. The column was washed with 50 ml TN buffer containing 15 mM imidazole and then the His_6_-MBP-TxIA fusion protein was eluted with 3 × 10 ml (each time 30 min) TN buffer containing 400 mM imidazole. The flow-through was concentrated to 5 ml (Amicon Ultra 30K filter) and diluted with 5 ml TN buffer. TxIA peptide was liberated from the fusion protein by cleavage overnight at room temperature using ∼100 μg TEV protease. In order to maintain TEV protease activity, reduced/oxidized glutathione were added at a ratio of 1:5 w/w.

### Recombinant Peptide Purification by HPLC

The protease-cleaved samples were acidified with trifluoracetic acid (TFA), centrifuged (10 min at 6,762 *g*) and filtered (Millipore ultrafree MC 0.2 μm) to remove protein precipitates. Then 50% acetonitrile (ACN) was added to a final concentration of 5% ACN and the peptides were separated on a C18 semi-preparative reversed-phase (RP) HPLC column (Vydac protein/peptide C18 column Cat# 218TP1010) on a Shimadzu Prominence HPLC system (Shimadzu, Rydalmere, NSW, Australia). The following linear gradients of solvent B (90% ACN, 0.043% TFA in water) in solvent A (0.5% TFA in water) were used at a flow rate of 3 ml/min: 5% B for 10 min, then 5–35% B for 30 min followed by 35–80% B over 5 min and 80% B for another 7 min. Absorbance was determined at 214 and 280 nm and collected fractions were lyophilized and stored at −20°C. The fractions containing the correct peptide masses as determined by matrix-assisted laser desorption/ionization mass spectrometry (MALDI MS) were then subjected to another HPLC fractionation using a hydrophilic interaction liquid chromatography (HILIC) column. Samples were dissolved in 95% solvent B and injected into a VisionHT HILIC column (5 μm particle size, 150 mm × 4.6 mm; Grace, Columbia, MD, United States) at a flow rate of 1 ml/min. The same solvents as during the RP-HPLC fractionation were used with the following linear gradients: 95% solvent B for the first 3 min, then 95–75% B over 20 min. Absorbance was determined at 214 and 280 nm and the molecular masses of the peptides determined using MALDI MS.

### Analysis by Liquid Chromatography/Mass Spectrometry

Solvents used for LC/MS were of HPLC-grade. Recombinant peptide masses were determined by MALDI time-of-flight (TOF) MS using a 4700 Proteomics Bioanalyzer model (Applied Biosystems, Carlsbad, CA, United States). Peptides were dissolved in water and mixed 1:1 (v/v) with α-cyano-4-hydroxycinnamic acid matrix (7 mg/ml in 50% ACN, 5% formic acid) and mass spectra acquired in positive reflector mode. All reported masses are for the monoisotopic [M+H^+^] ions.

For analysis of the synthetic TxIA ribbon analog, the LC/MS system consisted of an Waters Alliance 2695 HPLC (Milford, OH, United States) coupled to a Micromass ZQ mass spectrometer (electrospray ionization mode, ESI+). All analyses were carried out using a Chromolith (Fontenay-sous-Bois, France) HighResolution RP-18e column (4.6 mm × 25 mm, 15 nm–1.15 μm particle size). A flow rate of 3 ml/min and a gradient of 0–100% solvent B over 2.5 min were used. Eluent A was water/0.1% formic acid while eluent B was ACN/0.1% formic acid. UV detection was performed at 214 nm. ESI mass spectra were acquired using a solvent flow rate of 200 μl/min. Nitrogen was used for both the nebulizing and drying gas. Data were obtained in a scan mode over the m/z range 100–1000 or 250–1500 in 0.7 s intervals. Fully folded synthetic ribbon TxIA was characterized using a Synapt G2-S high-definition MS system (Waters, Corp., Milford, MA, United States) equipped with an ESI source. Chromatographic separation was carried out at a flow rate of 0.4 ml/min on a Acquity H-Class ultrahigh performance liquid chromatography (UPLC) system (Waters, Corp., Milford, MA, United States), equipped with a Kinetex C18 100A column (100 mm × 2.1 mm, 2.6 μm particle size) from Phenomenex (France). The mobile phase consisted of water (solvent A) and ACN (solvent B) with both phases acidified by 0.1% (v/v) formic acid. Mass spectra were acquired in the positive ionization mode.

### Two-Electrode Voltage Clamp (TEVC) Electrophysiology

cDNAs encoding rat nAChR subunits were provided by J. Patrick (Baylor College of Medicine, Houston, TX, United States) and subcloned into the oocyte expression vector pNKS2 (Gloor et al., 1995). cRNA was synthesized with the SP6 mMessage mMachine Kit (Ambion, Austin, TX, United States) and adjusted to a concentration of 0.5 μg/μl. nAChR subunit RNAs were mixed in the ratios 1:1 (α3/β2) or 5:1 (α4/β2). *Xenopus laevis* (Nasco International, Fort Atkinson, WI, United States) oocytes were injected with 50 nl aliquots of cRNA (Nanoject automatic oocyte injector, Drummond Scientific, Broomall, PA, United States). Antagonist concentration-response curves were measured as described previously (Dutertre et al., 2005; Beissner et al., 2012) in ND96 (96 mM NaCl, 2 mM KCl, 1 mM CaCl_2_, 1 mM MgCl_2_, and 5 mM HEPES at pH 7.4). In brief, current responses to acetylcholine were measured at room temperature 1–6 days after cRNA injection and recorded at −70 mV using a Turbo Tec 05X Amplifier (NPI Electronic, Tamm, Germany) and Cell Works software. A standard concentration of 100 μM ACh or nicotine was used to activate the αβ combinations and the α7 nAChR, respectively. A fast and reproducible solution exchange (<300 ms) was achieved with a 50-μl funnel-shaped oocyte chamber combined with a fast vertical solution flow fed through a custom-made manifold mounted immediately above the oocyte. Agonist pulses were applied for 2 s at 4-min intervals. Following 1 min of perfusion directly after the agonist application, peptides were applied in a static bath for 3 min. IC_50_ values were calculated from a non-linear fit of the Hill equation to the data (Prism version 6.0; GraphPad Software, Inc., San Diego, CA, United States). Data are presented as means ± SEM from at least three oocytes.

### Chemical Synthesis of Ribbon and Native α-TxIA

ACN, TFA, dimethylformamide (DMF), *N*,*N*-diisopropylethylamine (DIEA), triisopropylsilane (TIS), dichloromethane (DCM), piperidine and other reagents were obtained from Sigma-Aldrich (St. Louis, MI, United States) or Merck (Darmstadt, Germany) and used as supplied. Fmoc L-amino acid derivatives and 1[*bis*(dimethylamino)methylene]-1H-1,2,3-triazolo[4,5-b]pyridinium 3-oxid hexafluorophosphate (HATU) were purchased from Iris Biotech (Marktredwitz, Germany). AmphiSpheres 20 HMP resin (0.6 mmol/g) was purchased from Agilent Technologies (Les Ulis, France). The side chain protecting groups for amino acids are t-Bu for Asp and Ser; Acm for Cys_9,14_; Trt for Cys_1,15_ and Asn; Pbf for Arg. Ribbon α-TxIA was manually synthesized using the Fmoc-based solid-phase peptide synthesis technique on a VWR (Radnor, PA, United States) microplate shaker. All Fmoc amino acids and HATU were dissolved in DMF to reach 0.5 M. Chain elongation was performed step by step using 0.2 mmol of AmphiSpheres 20 HMP resin. Fmoc deprotection was performed with 20% piperidine in DMF two times, each for 1 min at room temperature, then the resin was washed three times with DMF. Each Fmoc-protected amino acid (5 eq.) was coupled twice in the presence of HATU (5 eq.) and DIEA (10 eq.) in DMF at room temperature for 2 min. After completion of the coupling reaction, the resin was sequentially washed twice with DMF. Cleavage of peptide from the resin and removal of side-chain protecting groups were carried out using 20 ml of a solution containing TFA/TIS/H_2_O (95:2.5:2.5, v/v/v) for 3 h at room temperature. After the resin was removed by filtration and washed with DCM, the DCM and TFA were removed under vacuum and cold diethyl ether was added to precipitate the peptide. Crude peptide was purified by preparative RP-HPLC, and its purity determined using LC/ESI-MS. Trt groups were removed during the cleavage step while the Acm protective groups are resistant to cleavage conditions. A two-step oxidation procedure was then carried out. Briefly, the first disulfide bridge was formed from free cysteines with the use of 2,2′-dithiopyridine (DTP) (Maruyama et al., 1999; Giribaldi et al., 2018) and then the second disulfide bridge was formed by concomitant deprotection and oxidation of the Acm groups with the use of iodine (Gyanda et al., 2013). The fully folded peptide was purified by analytical RP-HPLC and purity confirmed by high-resolution mass spectrometry During the purification process, the ribbon form of α-TxIA appears as a broad peak on analytical RP-HPLC (containing unseparable major and a minor peaks), which is possibly due to slow *cis*–*trans* interconversion of the proline residues.

Globular TxIA was synthesized as described (Dutertre et al., 2007).

### Synthetic Peptide Purification

Preparative RP-HPLC was run on a Gilson PLC 2250 HPLC system (Villiers le Bel, France) using a preparative column (Waters DeltaPak C18 Radial-Pak Cartridge, 100 Å, 40 mm × 100 mm, 15 μm particle size, flow rate 50.0 mL/min). Buffer A was 0.1% TFA in water, and buffer B was 0.1% TFA in ACN. Fully folded synthetic TxIA ribbon was purified on a UltiMate 3000 UHPLC system (Thermo Fischer Scientific) using an Kinetex C18 100 A column (100 mm × 2.1 mm, 2.6 μm particle size) from Phenomenex (France). Buffer A was 0.1% formic acid in water, and buffer B was 0.1% formic acid in ACN.

### NMR Spectroscopy Analysis

#### Secondary Shifts of Recombinant Peptides

Peptides were dissolved in 90% H_2_O/10% D_2_O (Cambridge Isotope Laboratories) at a concentration of 1 mg/ml and pH ∼3.6. Spectra were recorded on a Bruker Avance 600 MHz spectrometer at 7°C and referenced to 4,4-dimethyl-4-silapentane-1-sulfonic acid (DSS) at 0 ppm. Standard Bruker pulse programs were used for all two-dimensional spectra. Excitation sculpting with gradients was used to achieve water suppression for TOCSY and NOESY experiments (Hwang and Shaka, 1995). NMR experiments included TOCSY (Braunschweiler and Ernst, 1983) using a MLEV-17 spin lock sequence with a 80 ms mixing time, NOESY (Jeener et al., 1979) with a 200 ms mixing time, ^1^H- ^13^C HSQC (Palmer et al., 1991) and ^1^H-^15^N HSQC (Palmer et al., 1991). Spectra were recorded with 4096 data points in the F2 dimension and 512 increments in the F1 dimension for TOCSY, and NOESY experiments, 2048 × 240 for ^1^H-^13^C HSQC and 2048 × 128 for ^1^H-^15^N HSQC data points in the F2 dimension and increments in the F1 dimension, respectively. The t1 dimension was zero-filled to 1024 real data points, and the F1 and F2 dimensions were multiplied by a sine-squared function prior to Fourier transformation. All spectra were processed using TopSpin 2.1 (Bruker) and assigned with CcpNmr Analysis (Vranken et al., 2005) using the sequential assignment protocol (Wüthrich, 1986). Secondary shifts were calculated using the random coil values reported by Wishart et al. (1995).

#### Structure of Synthesized Ribbon TxIA

A 2D NOESY spectrum was acquired with a mixing time of 200 ms at 7°C and interproton distance constraints were calculated from the relative intensities of NOE cross-peaks. Predictions of ϕ and ψ backbone angles were performed with TALOS-N (Shen and Bax, 2013). Distance restraints for the disulfide bonds defined by the regioselective synthesis were used in the structure calculations. The ANNEAL function in CYANA was used to perform 10,000 steps of torsion angle dynamics to generate an initial ensemble of 100 structures from which the 20 structures with the lowest penalty function values were chosen for analysis. Several rounds of structure calculations were performed to resolve distance and angle constraint violations. Using protocols from the RECOORD database (Nederveen et al., 2005), an ensemble of 100 structures was subsequently calculated with CNS (Brünger et al., 1998) using the force field distributed with Haddock 2.0 (Dominguez et al., 2003) and further refined in a water shell (Linge and Nilges, 1999). A set of 20 structures with the lowest energy and no NOE violations greater than 0.2 Å or dihedral-angle violations greater than 3° was selected for MolProbity analysis (Williams et al., 2018). The structures were visualized and figures generated using MOLMOL (Koradi et al., 1996).

### Molecular Modeling

Molecular models of the complexes between the ligand-binding domain of α7 nAChR and conotoxins were prepared by homology modeling and then refined using either molecular dynamics (MD) simulations or the ToxDock method (Leffler et al., 2017). The molecular models are provided along the manuscript in Supplementary Material Data Sheet 1. Homology models were constructed using Modeler 9v18 (Sali and Blundell, 1993) using three templates: (i) the NMR solution structure of ribbon TxIA (this study); (ii) the α4/α4 interface of the cryo-electron microscopy structure of the human α4β2 nAChR (PDB 6CNK) (Walsh et al., 2018); and (iii) the crystal structure of the complex between globular conotoxin [A10L]TxIA and the *Aplysia californica* acetylcholine binding protein (AChBP, PDB 2UZ6) (Dutertre et al., 2007). A 100 models were generated for each system and the model with the lowest DOPE score (Shen and Sali, 2006) was selected for further refinement.

#### Refinement With MD Simulations

Homology models of the interaction of ribbon deamidated TxIA, [R5D] ribbon TxIA, and [R5N] ribbon TxIA with rat α7 nAChR were obtained by homology modeling as described above. These initial models were then minimized and subjected to MD simulations in Gromacs 2018 using the Ambr99SB-ILDN forcefield (Lindorff-Larsen et al., 2010). The systems were embedded in a cubic box of 11 nm × 11 nm × 9 nm and solvated with ∼32,000 water molecules and several sodium ions to neutralize the systems. The system was then energy minimized using 10,000 steps of steepest gradient and submitted to an equilibration protocol during which position restraints on the receptor and toxin were progressively released; the constraints on the toxin atoms were released first over 6 ns, and the constraints on the receptor then over 2.4 ns. The systems were then simulated unrestrained for 50 ns. Long-range electrostatic interactions were computed using particle mesh Ewald with default parameters in Gromacs. All bonds involving hydrogens were constrained with the LINCS algorithm, and the time step of the simulation was set to 2 fs. The V-rescale thermostat (Bussi et al., 2007) was used to maintain the temperature at 27°C and the pressure was maintained at one atmosphere using the Berendsen isotropic pressure coupling. The short range electrostatic and Van der Waals cutoffs were both set at 1 nm. The binding free energy of each system was then computed using the MMPB/SA method (Miller et al., 2012) as implemented in Amber18 by analyzing 10 frames extracted every ns from the last 10 ns of the unrestrained simulation.

#### ToxDock Refinement

The ToxDock (Leffler et al., 2017) refinement and energy computations were carried out using the online server Rosie (Lyskov et al., 2013). Briefly, the Rosetta fast relax protocol (Tyka et al., 2011) was used to generate 200 alternative models representing small structural fluctuation of the complex. The 50 models with lowest Rosetta total score were submitted for a FlexPepDock refinement (Raveh et al., 2010), generating 500 alternative binding modes of the peptide bound to the receptor and computing the reweighted docking Rosetta score. The average of the 25 lowest reweighted score was taken as the relevant to the binding score of the complex, as previously described (Leffler et al., 2017). The Talaris2013 energy function was used for all Rosetta computations (Leaver-Fay et al., 2013).

## Results

### Efficient Production of Recombinant TxIA Analogs

**FIGURE 1:**
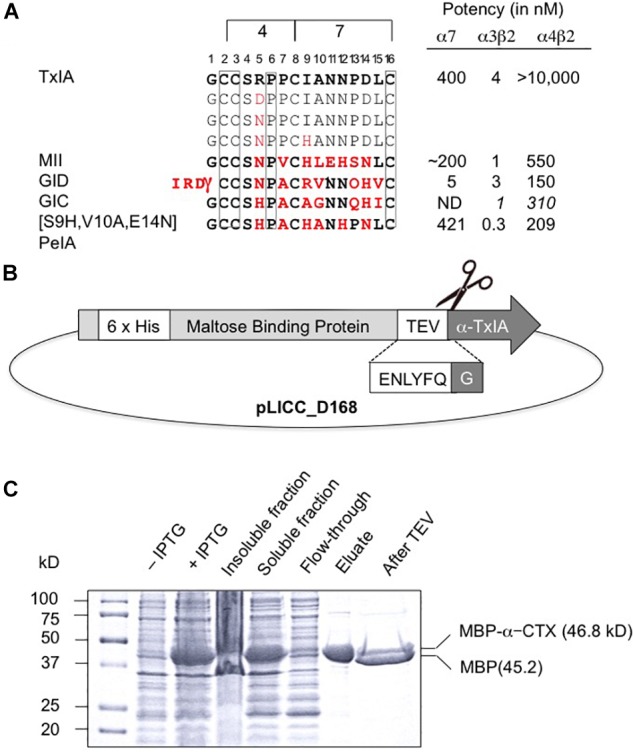
Recombinant production of TxIA. **(A)** Amino acid sequence alignment of native and mutant analogs of TxIA with α-conotoxins that show some affinity to α4β2 nAChRs, and their respective IC_50_ values (for references see, Dutertre et al., 2017). Italics indicate values at human nAChR isoforms. Conserved cysteine residues are shown in boxes. The number of residues between the cysteines defines the 4/7 α-conotoxin subfamily. Residues in MII, GID, GIC, PeIA that differ from TxIA are shown in bold red. Mutated TxIA residues in position 5 and 9 are also highlighted in red. IC_50_ values refer to studies using *Xenopus laevis* oocytes. **(B)** Schematic representation showing the design of the pLic-MBP plasmid used for periplasmic expression of disulfide-rich peptides in *Escherichia coli*. The construct encodes a His_6_ tag for affinity purification, followed by a MBP fusion tag for solubility and a TEV protease recognition site between the MBP and the TxIA sequences. **(C)** Coomassie Blue stained SDS-PAGE gels showing samples of the fusion construct obtained during different steps of recombinant peptide production and purification. The difference in molecular mass between the concentrated eluate before (second last lane) and after (last lane) TEV protease cleavage indicates cleavage of TxIA from the His_6_-MBP.

Like most characterized 4/7 α-conotoxins, TxIA preferentially targets α7 and α3β2 nAChRs with nanomolar potency but has no detectable activity at the α4β2 subtype (Dutertre et al., 2007). Whereas some α3β2-selective conotoxins such as MII (Azam and McIntosh, 2009) also have high potency at the closely related α6β2 receptor, no conotoxin that selectively targets the α4β2 nAChR has been yet identified, and only a few 4/7 α-conotoxins, such as MII (Cartier et al., 1996), GID (Nicke et al., 2003), and GIC (McIntosh et al., 2002) have been shown to block this receptor, albeit at high nanomolar or micromolar concentrations. Therefore, one aim of this study was to identify amino acid side chains that could prevent conotoxin binding to α4β2 receptors by investigating if TxIA could be modified to obtain a conotoxin with at least weak activity at α4β2. To this end, we compared the amino acid sequence of TxIA with those of MII, and GID and selected two positions, 5 and 9, in which we substituted the amino acid side chains (Figure 1A). Position 5 was previously shown to be important for MII binding to α3β2 nAChRs (Everhart et al., 2004).

**FIGURE 2:**
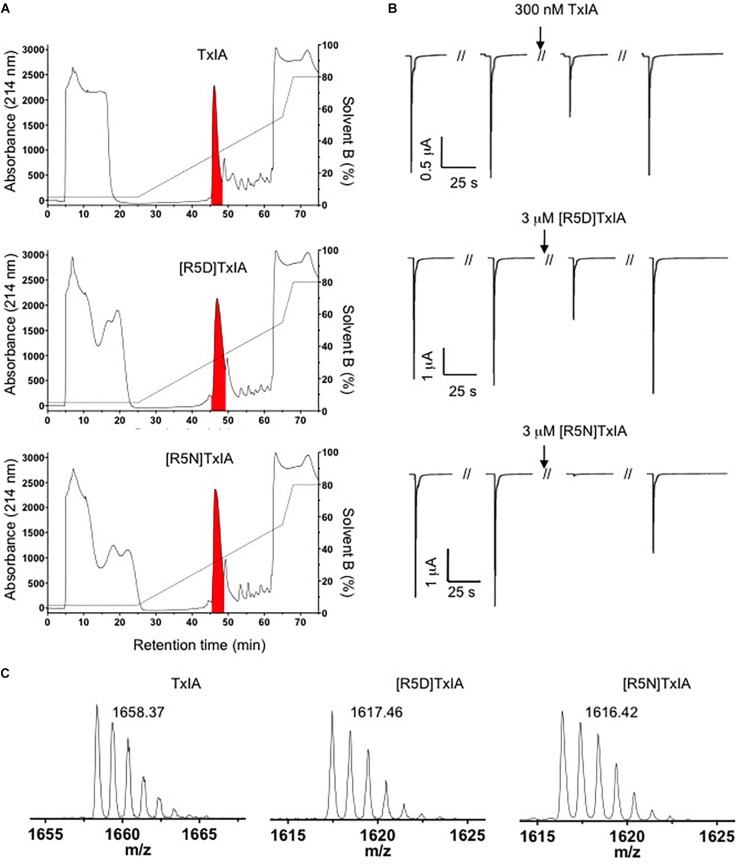
Purification of TxIA and mutant (R5D, R5N) analogs. **(A)** RP-HPLC chromatograms showing first step RP-HPLC fractionation with the peaks highlighted in red containing the correct molecular mass as determined by MALDI MS. **(B)** Antagonistic activity of the purified toxin peak (i.e., the red peak from **A**) at α3β2 nAChRs. Oocytes were clamped at –70 mV and 100 mM ACh were used to activate α3β2 nAChRs. **(C)** MALDI MS traces after second step HILIC fractionation of the purified TxIA and analogs (red colored fractions in Supplementary Figure S2) indicate the monoisotopic molecular masses of the purified toxins. All reported masses are for [M + H]^+^ ions.

The DNA sequences corresponding to TxIA and analogs were optimized for expression in *E. coli* and fused N-terminally via a TEV protease cleavage site to MBP (Figure 1B). Although the consensus TEV protease cleavage site is ENLYFQ/S, the protease also efficiently cleaves at ENLYFQ/G (Kapust et al., 2002), which has the advantage that the N-terminal glycine residue that is present in most α-conotoxins can be used and remains after cleavage. Expression of the TxIA analogs in form of a His_6_-MBP fusion protein was done to target the construct to the *E. coli* periplasmic space where the disulfide bond folding machinery of the bacterium (i.e., the Dsb proteins) is located and the oxidative environment is favorable for conotoxin folding and disulfide bond formation. Upon transformation of *E. coli* with these constructs and induction with IPTG, the respective fusion proteins represented the dominant proteins produced and they could be successfully purified and cleaved as indicated by the size shift of the MBP fusion construct (Figure 1C and Supplementary Figure S1A). Further purification by RP-HPLC revealed one dominant peak for each of the TxIA analogs (Figure 2A and Supplementary Figure S1B). The corresponding fractions contained the calculated mass of the fully oxidized peptides (Figure 2C) and turned out to represent the sole functional fractions when tested by TEVC electrophysiological analysis on α3β2 nAChRs expressed in *Xenopus laevis* oocytes (Figure 2B and Supplementary Figure S1C). The obtained yields were 1–2 mg of conotoxin per liter of culture. An additional HILIC-HPLC purification step was employed (Supplementary Figure S2) to obtain pure recombinant peptides as determined using MALDI-MS (Figure 2C and Supplementary Figure S2). These purified peptides were then used for preparation of concentration response curves (see below). The double mutant [R5N,I9H] was not further analyzed.

### Electrophysiological Analysis of Recombinant TxIA Analogs Reveals Strongly Reduced Potency

**FIGURE 3:**
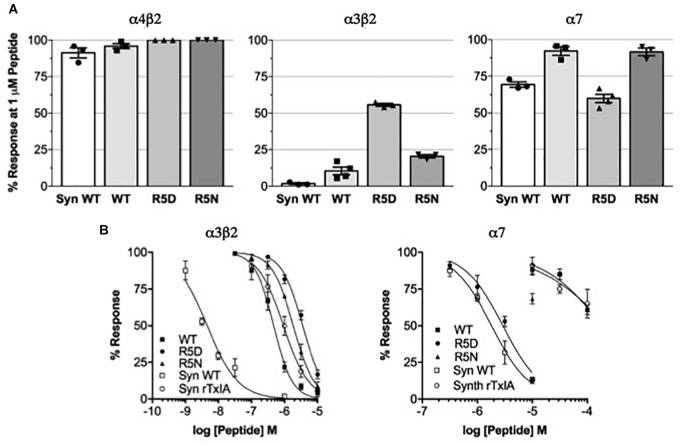
Potency comparison of synthesized amidated and expressed non-amidated TxIA on nAChR subtypes. **(A)** Reduction of control responses activated by 100 μM ACh or nicotine (in case of α7 nAChR) by 1 μM synthetic (syn) or recombinant TxIA (rec = wild type; R5D and R5N amino acid substitutions) at the indicated nAChR subtypes expressed in *Xenopus* oocytes and measured using TEVC electrophysiology. **(B)** Concentration-response curves for synthetic and recombinant TxIA at α3β2 (left) and α7 (right) nAChRs. Error bars represent the SEM from at least three recordings [except for high (>10 μM) concentrations of synthetic peptides, where not enough material was available]. Oocytes were clamped at –70 mV. 100 μM ACh or 100 μM nicotine were used to activate α3β2 and α7 receptors, respectively.

We compared the potencies of the repurified expressed peptides TxIA, [R5D]TxIA, and [R5N]TxIA with synthetic TxIA at oocyte-expressed α3β2, α4β2, and α7 nAChRs. Unlike the *E. coli*-expressed α-conotoxins, but like native TxIA, the synthetic TxIA was C-terminally amidated. Not only were none of the analogs active at the α4β2 nAChR but all recombinant peptides showed a marked drop in potency compared to synthetic TxIA (Figure 3A). Preparation of full concentration-response curves revealed that in comparison to synthetic TxIA, which yielded IC_50_ values of ∼5 nM and 2 μM at α3β2 and α7 nAChRs, respectively, the recombinant TxIA was about 100- and 50-fold less active at α3β2 and α7 nAChRs (Figure 3B and Table 1). Interestingly, the potency decrease at the α7 receptor was almost completely reversed when the arginine residue in position 5 was substituted by an aspartic acid residue, but not when an asparagine residue was introduced in this position. Consequently, whatever caused the decrease in activity of the recombinant TxIA, lack of amidation, proline isomerization, and/or misfolding of the peptide, could be compensated by changes in the primary structure.

**Table 1:** IC_50_ values and Hill coefficients (*n*_H_) for synthetic TxIA and recombinant TxIA, [R5D]TxIA, and [R5N]TxIA at rat α3β2 and α7 nAChRs.

	α3β2		α7	
	IC_50_ (nM)	*n*_H_	IC_50_ (nM)	*n*_H_
Synthetic	4.6	−0.93	1812	−1,23
TxIA	(3.6 to 5.8)	(−1.15 to −0.71)	(1555 to 2111)	(−1,52 to −0,99)
Synthetic	928	−1.14	*≈ 200.000*	ND
rTxIA	(810 to 1064)	(−1.3 to −0.98)		
Recombinant	470	−1.54	*≈ 200.000*	ND
TxIA	(432 to 512)	(−1.73 to −1.35)		
Recombinant	3543	−1.38	2915	−1.24
[R5D]TxIA	(3166 to 3965)	(−1.58 to −1.17)	(2406 to 3532)	(−1.53 to −0.96)
Recombinant	1834	−1.36	ND	ND
[R5N]TxIA	(1704 to 1974)	(−1.49 to −1.24)		

*Numbers in brackets are 95% confidence intervals. ND means not determined. Italic numbers are estimates.*

### Structural Analysis of Recombinant TxIA Analogs Confirms the Preferential Formation of Ribbon Isomers

**FIGURE 4:**
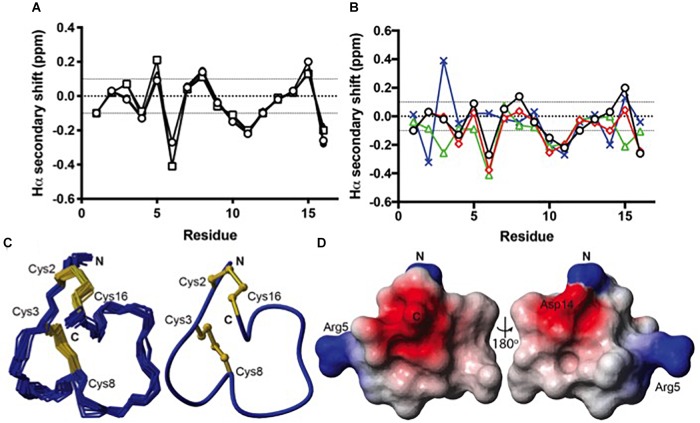
Structural analysis of the ribbon disulfide isomers of TxIA and analogs. **(A)** Hα secondary shift values for recombinant TxIA (open circles), [R5D]TxIA (squares), [R5N]TxIA (triangles), and synthetic ribbon TxIA (closed circles) at 7°C. Please note that due to the identity of the peptides, the secondary shifts overlap and most data points (filled circles) are hardly visible **(B)** A comparison of the Hα secondary shifts for ribbon TxIA (black) with the Hα secondary shifts of ribbon (red), globular (green) and beads (blue) isomers of Pn1.2. The values for ribbon TxIA most closely match those of ribbon Pn1.2, especially in the N-terminal region. **(C)** Left panel: ensemble of the 20 lowest energy structures for ribbon TxIA. The peptide backbone is in blue and the disulfides are in gold. Right panel: the mean structure of ribbon TxIA calculated from the ensemble. The structure is shown in ribbon format with disulfide bonds in ball and stick. The N- and C-termini are indicated and the cysteines are labeled. **(D)** Electrostatic surface of ribbon TxIA (PDB ID 6OTA) highlighting the negatively charged regions (red) corresponding to the C-terminal acid and Asp14, and the positively charged regions (blue) corresponding to the N-terminus and the side chain of Arg5.

Because all of the recombinant peptides showed at least some potency at α3β2 nAChRs and peptides in the other fractions turned out to be inactive or less active, we assumed that the dominant peaks corresponded to the native (i.e., globular) conformation of the peptides. However, because of the strong potency decrease that is clearly higher than the 2- or 5-fold potency reduction of non-amidated AnIB analogs at α3β2 and α7 nAChRs, respectively (Loughnan et al., 2004) we considered the possibility of non-native disulfide linkage in the *E. coli*-expressed TxIA analogs. To test this hypothesis, NMR structural analysis was performed. The three expressed TxIA analogs yielded well-dispersed ^1^H NMR resonances, implying that they adopt ordered structures in solution. These peptides were further analyzed using two-dimensional NMR so that the data could be compared to published data for Pn1.2, which has a related sequence and where NMR data for each of the disulfide isomers is available (Carstens et al., 2016). To assign the spectra, Hα-NH_i+1_ connectivities obtained from NOESY spectra were used in the sequential assignment of individual spin systems determined from the TOCSY spectra. For all of the TxIA analogs, sequential Hα-NH_i+1_ connectivities were observed for the entire peptide chain, except at the Pro residues that lack backbone amide protons. Analysis of the NOE data and the Cδ chemical shifts of Pro7 indicated that this proline is in the *cis* confirmation (Schubert et al., 2002). Analysis of secondary shift data can supply information on secondary structural elements of peptides (Wishart et al., 1991), and is useful for comparing structural frameworks in disulfide-rich peptides. Figure 4B shows the Hα secondary shifts for TxIA and compares these values with previously published secondary shifts for the three possible disulfide isomers of Pn1.2. The Hα secondary shifts for the TxIA most closely resemble those of the ribbon isomer of Pn1.2, especially in the N-terminal region, which suggests that the expressed TxIA is in the ribbon form. Furthermore, a comparison of the secondary shifts for each expressed TxIA analog (Figure 4A) and synthetic ribbon TxIA (see below and Supplementary Figure S3C) reveals that they are almost identical and all four peptides form the same disulfide isomer. Most of the secondary shifts for the TxIA analogs vary within the ± 0.1 ppm range that is consistent with a random coil structure, however residues 9–11 have secondary shift values more negative than −0.1 ppm, suggesting this region has some helical character. Our structural and functional characterization of the TxIA analogs is in agreement with data from Wu et al. (2014), where an 80-fold potency reduction (IC_50_ of 5.4 to 430 nM) of synthesized ribbon TxIA was observed at α3β2 nAChRs, which corresponds well to the 100-fold reduction found in our study. Importantly, this analysis further confirmed the purity of the expressed toxins as similar retention times for globular and ribbon TxIA have been described (Wu et al., 2014).

### Solution Structure and Potential Binding Mode of the Ribbon Isomer of TxIA

α-Conotoxins are generally considered to be weakly active or inactive if they are folded in non-native conformations such as “ribbon” or “bead” disulfide isomers. However, in case of the 4/6 conotoxin AuIB, the ribbon isomer turned out to be more potent at α3β4 nAChRs than the globular form and seemed to bind to the orthosteric binding site (Grishin et al., 2010). Likewise, the ribbon form of the 4/3 α-conotoxin ImII was found to compete with binding of its natively folded isomer at α7 nAChRs and also bound to an additional binding site at *Torpedo* nAChRs (Kasheverov et al., 2009). For AuIB, a binding mode has recently been defined in detail (Wu et al., 2018). However, no ribbon 4/7 α-conotoxin structure has been determined to date (Kaas et al., 2012). Inspired by the fact that ribbon isomers of some conotoxins can competitively block ACh binding and that TxIA potency at the α7 nAChR could be improved by the substitution R5D, we set out to determine the structure of the ribbon conformer of TxIA and its binding mode at α7 nAChR. To this end, a non-amidated ribbon isomer of TxIA was synthesized (Supplementary Figure S3) by a two-step oxidation method and its NMR structure calculated using CYANA 3.0 (Güntert et al., 1997) and refined in CNS (Brünger et al., 1998) using TALOS-N (Shen et al., 2009) derived dihedral angle restraints, and hydrogen bond restraints derived from D_2_O exchange experiments.

**Table 2:** Structural statistics for the final ensemble of 20 structures for the ribbon isomer of TxIA with the highest overall MolProbity score.

Energies (kcal/mol)	
Overall	−475.3 ± 3.1
Bonds	6.51 ± 0.67
Angles	20.11 ± 2.48
Improper	9.21 ± 2.26
Van der Waals	−36.82 ± 3.04
NOE	0.02 ± 0.01
cDih	0.98 ± 0.67
Dihedral	65.78 ± 0.89
Electrostatic	−541 ± 6.3
**MolProbity statistics**	
Clashes (>0.4 Å/1000 atoms)	14.3 ± 4.66
Poor rotamers	0.05 ± 0.22
Ramachandran outliers (%)	0.0 ± 0.0
Ramachandran favored (%)	85.5 ± 6.19
MolProbity score	2.29 ± 0.31
Residues with bad bonds	0.00 ± 0.00
Residues with bad angles	0.00 ± 0.00
**Atomic RMSD (Å)**	
Mean global backbone	0.58 ± 0.26 (residues 1–16)
Mean global heavy	1.19 ± 0.37 (residues 1–16)
**Distance Restraints**	
Intraresidue (| i-j| = 0)	35
Sequential (| i-j| = 1)	44
Medium range (1 < | i-j| < 5)	16
Long range (| i-j| > 5)	8
Hydrogen bonds	3
Total	106
**Dihedral angle restraints**	
ϕ	11
ψ	8
χ^1^	9
Total	28
**Violations from experimental restraints**	
Total NOE violations exceeding 0.3 Å	0
Total dihedral violations exceeding 3.0^0^	0

The structural statistics for the ensemble of the 20 lowest energy structures for each isomer are shown in Table 2, and the structural ensemble of the 20 representative structures are shown in Figure 4C (left). The mean structure of the ribbon isomer of TxIA (Figure 4C, right), although well-defined (backbone RMSD = 0.58 ± 0.3 Å), possesses no defined secondary structure elements. However, several of the structures in the ensemble have a 3_10_-helical segment spanning residues Cys8 to Asn11, which is generally consistent with the Hα secondary shift data. Furthermore, loop 1 of ribbon TxIA (Cys2–Cys8) is well-defined (backbone RMSD = 0.21 ± 0.07 Å) and resembles loop 1 of globular TxIA, but loop 2 (Cys8–Cys16) is less well-defined (backbone RMSD = 0.48 ± 0.29 Å) and adopts a conformation that is distinct from loop 2 of globular TxIA. Analysis of the electrostatic surface features in MOLMOL reveals negatively charged regions on opposite faces, corresponding to the C-terminal acid and the sidechain of Asp14, and a positively charged region due to the sidechain of Arg5 and N-terminus (Figure 4D). This structure was subsequently used to probe the binding mode in a MD-refined model and a model based on the recently defined ToxDoc application.

### Molecular Modeling

#### Binding Modes in the ToxDock- and MD-Refined Models

**FIGURE 5:**
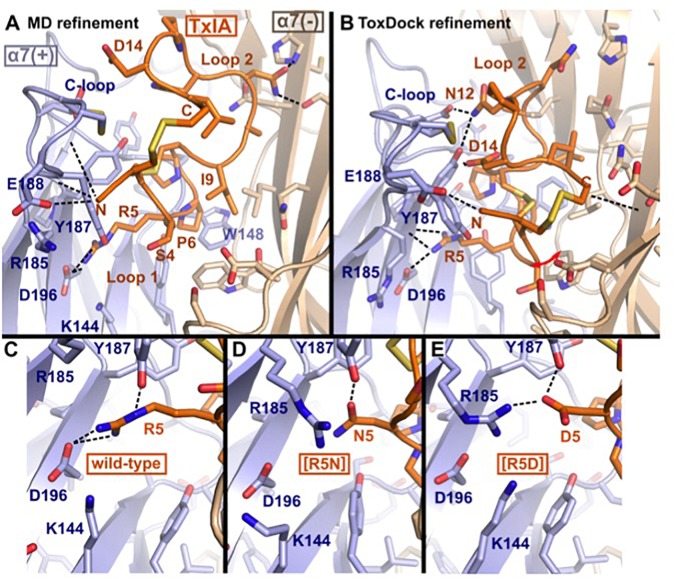
Molecular models of the binding modes of ribbon TxIA at the α7 nAChR: **(A)** MD-refined molecular model, **(B)** ToxDock refined molecular model, and interaction of position 5 in the MD-refined model of **(C)** ribbon TxIA, **(D)** ribbon [R5N]TxIA, or **(E)** ribbon [R5D]TxIA. The last frame of the 50 ns MD simulation is shown in **(A)**. The model with the lowest re-weighted Rosetta score is shown in **(B)**. The backbone of the receptor and peptides are shown using cartoon representation [blue and wheat color for the principal (+) and complementary (–) subunits, respectively], the side chains of the toxins are shown as sticks (orange) and the side chain of receptor residues located at less than 5 Å from the toxin are shown in stick representation. Hydrogen bonds are indicated by dashed black lines.

The binding modes of ribbon TxIA at the α7 nAChR generated by MD simulations and by ToxDock are globally similar in terms of the orientation of the toxin in the binding site, with some molecular interactions shared between the two models but also a range of interactions that are different (Figure 5A,B). Pro6 in Loop 1 of wild-type or variant ribbon TxIA occupies a similar position in the models as P6 of the globular TxIA in the TxIA/AChBP experimental structure. P6 is an important determinant of the interaction of α-conotoxins as it is embedded in the aromatic box, which is at the bottom of the orthosteric binding site and is recognized by acetylcholine. Another important conserved feature of the interaction of globular α-conotoxin with AChBP is the stacking between the vicinal disulfide bridge of the C-loop with the first disulfide bond of the α-conotoxin. The MD-refined model displays this interaction but not the ToxDock-refined model in which the side chain of D14 stacks between the two disulfide bonds. This is interesting because the conformation of toxin in the two models is globally preserved (the backbone RMSD of the toxins in the two models is only 1.5 Å). The binding mode in the ToxDock model is slightly tilted compared to the MD model, and this leads to a change in reorientation of the N-terminus (from G1 to S4) and of Loop 2 in general. The conformation of the wild-type and variants in the context of the binding site was highly stable, with a backbone RMSD < 1.5 Å and on average 1 Å from the initial homology model. The binding modes of the toxins were also similarly stable, with Loop 1 being highly rigid and Loop 2 undergoing larger, albeit small, conformational fluctuation. The binding mode of the ToxDock model was not sampled during the MD refinement, and they should therefore be considered as two hypothetical binding modes. The location and interaction of position 5 in the models of the wild-type TxIA are similar, and the interactions of this position in TxIA and variants will now be described using the MD-refined models (Figure 5C–E). Supplementary Figure S4 describes the interaction as suggested in the ToxDock-refined models.

#### Molecular Interactions of Position 5

TxIA R5 is located in Loop 1 and establishes a salt-bridge with α7 D196 in the wild-type model, similar to the interaction of globular TxIA R5 with AChBP D197 in the TxIA/AChBP crystal structure. The model also suggests the proximity of two principal subunit basic residues, α7 R185 and K144, which could create an unfavorable electrostatic environment for Arg 5. In contrast, the model of ribbon [R5D]TxIA shows that D5 establishes a salt bridge with α7 R185. In the ToxDock model of [R5D]TxIA, D5 also forms a salt-bridge but with α7 K144. Position 5 therefore seems to be better suited for a negatively charged residue than a basic residue. Asn and Asp are isosteric, but Asn is neutral whereas Asp is negatively charged. In contrast to [R5D]TxIA, the mutant displaying an Asn at position 5, i.e., [R5N]TxIA, has similar activity to the parent peptide, strongly suggesting that the charge of the side chain at position 5 drives the increase in activity at the α7 nAChR (Figure 5D,E). This observation also suggests that the charge interactions of TxIA R5 with the complementary subunit balanced out and do not contribute to affinity. Interestingly, this analysis suggests that an R5D substitution of the globular TxIA would increase affinity for the α7 nAChR.

#### Mutational Energy Predictions

The interaction energies predicted by ToxDock were similar for the three complexes, with the interaction energy of wild-type ribbon TxIA in Rosetta units (RUs) being −592 for TxIA, −594 for [R5D]TxIA, and −595 for [R5N] TxIA. The mutational energies predicted by MMPB/SA suggest that the [R5N] mutant should have increased affinity by −4 kcal/mol compared to the parent peptide, while the [R5D] substitution was predicted to decrease affinity by 13 kcal/mol. None of the energy predictions therefore correlated with the experimental IC_50_ values, possibly because the main determinants of the affinity change involve evaluating the interaction of salt bridges in a partially solvated environment in the vicinity of several oppositely charged residues, which is challenging. Current force fields still have difficulty in reproducing the strength of solvated salt bridges (Ahmed et al., 2018). In addition, the estimation of binding energy is highly dependent on the accuracy of the three-dimensional structures of the complex. The incomplete consensus between the ToxDock- and MD-refined models suggest that these binding modes of ribbon α-conotoxins are not accurate enough for predicting the impact of mutations using score or energy computations. The determination of an experimental structure of a ribbon conotoxin bound to either AChBP or a nAChR should dramatically increase our ability to predict the interaction of ribbon conotoxins and the impact of mutations.

## Discussion

Here we describe for the first time the production of a recombinant α-conotoxin (4/7 α-conotoxin TxIA and three analogs) by expression in the periplasm of *E. coli*. We characterized the structure of three of these recombinant toxins and determined that, contrary to expectation, they adopt a ribbon conformation, which is not the dominant disulfide connectivity obtained during random oxidation of synthetically produced α-conotoxins. Ribbon TxIA experienced a significant decrease in activity at the α3β2 and α7 nAChRs, and we discovered that the mutant [R5D]TxIA could rescue the activity at the α7 nAChR at a similar level to that of globular TxIA, possibly because the location of position 5 in the α7 binding site is more electropositive.

### Expression of α-Conotoxins in Bacteria

So far, three α-conotoxins, the 4/7 α-conotoxins Vc1.1, LvIA, and TxIB, have been recombinantly expressed (Singer et al., 2012; Zhu et al., 2016; Yu et al., 2018). Vc1.1 was expressed in the non-pathogenic *Salmonella enterica* serovar Typhimurium strain LT2. It was fused via a TEV protease cleavage site to a flagellar secretion substrate FlgM, enabling export of recombinant non-flagellar peptides through the flagellum and into the surrounding medium (Singer et al., 2012). No functional or structural data of *Salmonella*-expressed Vc1.1 were provided in that study.

LvIA was expressed in tandem repeats of various lengths in *E. coli* and purified from inclusion bodies which are generated during conventional recombinant protein expression in *E. coli* (Zhu et al., 2016). To allow chemical cleavage with cyanogen bromide, the individual peptide sequences were linked by methionine residues, which resulted in the N-terminal addition of a methionine residue to the cleaved peptides. Using this procedure and folding by air oxidation, a 18-fold decrease in activity from 9 to 160 nM was observed for the recombinant peptide compared to synthetic LvIA (Luo et al., 2014; Zhu et al., 2016). This decrease in activity was suggested to be due to the additional N-terminal methionine and/or the missing C-terminal amidation. The latter would be in agreement with the 2- and 5-fold potency reduction of non-amidated AnIB analogs at α3β2 and α7 subtypes respectively (Loughnan et al., 2004). However, the disulfide connectivity of the *E. coli*-expressed LvIA was not determined and our study of recombinant TxIA suggests that alternative disulfide connectivity could also explain the activity change.

In a more recent study from the same group (Yu et al., 2018), monomeric TxIB was expressed in *E. coli* as a ketosteroid isomerase (KSI)–TxIB(M)–His_6_–fusion protein. KSI was used to help stabilize the peptide in inclusion bodies and the insoluble KSI could be easily separated upon cleavage from the peptide. A C-terminal methionine residue (M) had to be added to allow subsequent release of the peptide from KSI and the His_6_-tag by cleavage with cyanogen bromide. Interestingly, the recombinant TxIB retained selectivity for α6β2 receptors and showed only a moderate twofold decrease in potency. Based on these properties, a globular fold was inferred because ribbon and bead isomers of TxIB were reported to be inactive.

Encouraged by reports on bacterial expression of other toxins and in an attempt to bypass the problem of aggregation in inclusion bodies, we exploited a system that allows expression of venom peptides in the form of periplasmic MBP fusion proteins. The idea of using periplasmic expression is to hijack the disulfide-bonding machinery in *E. coli* for producing natively folded heterologous peptides (Klint et al., 2013). Here, intramolecular disulfide bonds should form as the polypeptide chain exits the reductive environment of the bacterial cytoplasm and enters the oxidizing periplasmic environment. With a success rate of 75% based on the expression of 31 venom peptides (ranging from 17 to 76 residues in length with 2–6 disulfide bonds) from spiders, scorpions, sea anemone, and cone snails, this method mostly produces correctly folded peptides (Klint et al., 2013). However, the 25% of peptides that failed to express using this method indicate that the folding machinery in *E. coli* is not always a good surrogate for the mechanisms found in the venomous organisms from which the respective peptides were sourced. In the case of venom peptides from cone snails, the only other peptide that was tried (and failed) in this expression system was MVIA (Klint et al., 2013). In regard to TxIA, we could also not produce any native (globular) isomer, as all of the recombinant TxIA formed the ribbon isomer. In summary, for the only two *Conus* venom peptides that have been investigated so far, periplasmic expression in *E. coli* failed to produce the native disulfide-bond isomer. However, additional data with other *Conus* peptides is required to make a conclusion about the general suitability of this system for producing natively-folded cone snail venom peptides.

### Does the Primary Structure Affect the Folding?

One surprising finding was that all expressed variants eluted in one dominant peak. In contrast to the present results and in agreement with data on other α-conotoxins, chemically synthesized and randomly oxidized α-conotoxin TxIA folds in all three possible conformations, globular, ribbon, and beads (Wu et al., 2014). This suggests the presence of factors that aid the formation of ribbon isomers in the periplasm of *E. coli* in contrast to the venom gland of the cone snail, in which the globular isomer is formed. In that case, misfolding could be a general problem for *E. coli* expression of α-conotoxins. Alternatively, TxIA could have features in its amino acid sequence that favor formation of the ribbon fold, such as non-covalent interactions that stabilize the respective cysteine positioning. For example, a study by Kang et al. (2005) found that C-terminal amidation can cause a preferential (but not absolute) formation of the globular form of the 4/3-α-conotoxin ImI, supposedly by different hydrogen bonding interactions of the C-terminus. In this context, the potential effect of a Pro-Pro motif present in the first loop of TxIA instead of the more common Pro-Ala motif might also be important due to the structural constraints induced by proline residues. However, TxIB, which appeared to fold preferentially in the globular fold (Yu et al., 2018) has a first loop with amino acid sequence identical to [R5D]TxIA, which favored the ribbon conformation in our study. Thus, factors in loop 2 of 4/7 α-conotoxins would be more likely to affect the disulfide formation.

The inability to create post-translational modifications of α-conotoxins in *E. coli* (besides disulfide bonds) is potentially a problem as some of these modifications are important for activity in some conotoxins. Nevertheless, certain modifications such as tyrosine sulfation, glutamate carboxylation or C-terminal amidation had relatively minor effects in previous studies and might be dispensable for some toxins (Nicke et al., 2003; Loughnan et al., 2004). *In vitro* modifications such as C-terminal amidation, might also be feasible on purified recombinantly expressed toxins (Ul-Hasan et al., 2013; Zielinski et al., 2016).

### Structural Comparison of 4/7 α-Conotoxin Ribbon and Globular Isomers

**FIGURE 6:**
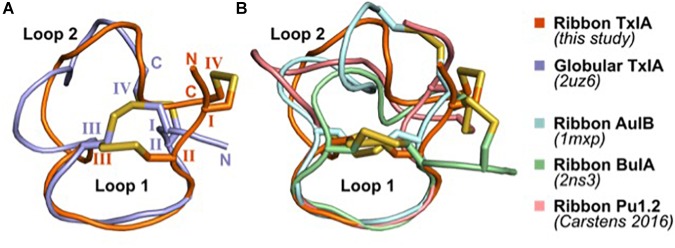
Comparison of the NMR solution structure of ribbon TxIA with **(A)** globular TxIA and **(B)** other ribbon contoxin structures. The first model from the NMR ensemble was used in this figure, with the caveat that the 20 models of ribbon AuIB display a high conformational variability of Loop 2. The peptides are shown in cartoon representations and the disulfide bonds are indicated as yellow sticks. The N- and C-termini are indicated in **(A)**, and the hemi-cystine residues are numbered using Roman numerals. The PDB identifier of the experimentally determined three-dimensional structures is indicated where available.

The solution structures of four ribbon α-conotoxins have been determined and are publicly available; these include the 3/5 α-conotoxin GI (PDB 1XGB) (Gehrmann et al., 1998), the 4/4 α-conotoxin BuIA (PDB 2NS3) (Jin et al., 2007), the 4/6 α-conotoxin AuIB (PDB 1MXP) (Dutton et al., 2002), and the 4/6 α-conotoxin Pu1.2 (Carstens et al., 2016). The structures of ribbon conotoxins are typically less well-defined than that of the corresponding globular isomers, with Loop 1 being relatively rigid whereas Loop 2 adopts multiple conformations, presumably indicating a greater flexibility (Figure 6). The four ribbon conotoxins with four residues in Loop 1, i.e., BuIA, rAuIB, Pu1.2, and TxIA, have a very similar conformation of Loop 1. Their Loop 2 conformations are different, reflecting the larger structural variability of Loop 2 in globular conotoxins (Akondi et al., 2014).

The ribbon isomers of α-conotoxins have naturally occurring structural analogs, the cysteine Framework X, with which they share two disulfide bridges in a ribbon isomer connectivity, a similar size, and four cysteine residues in similar pattern along the amino acid sequence (i.e., CC-C-C) (McIntosh et al., 2000). Loop 2 of cysteine framework X conotoxins contains only two amino acids, with the second residue of this loop being a hydroxyproline. The Loop 1 of framework X toxins has four residues, similar to the cysteine Framework I α-conotoxins, but it does not contain the Pro residue that is conserved in the α-conotoxins, and which occupies the orthosteric binding site of nAChRs as suggested by the crystal structures of complexes between α-conotoxins and AChBP (Lin et al., 2016). Framework X conotoxins are not active at nAChRs but rather target the neuronal noradrenaline transporter, and hence they are classified in the χ pharmacological family (Sharpe et al., 2001). Interestingly, the ribbon isomer of an α-conotoxin was even identified in a cone snail venom, indicating that for some α-conotoxins the ribbon isomer is also native (Safavi-Hemami et al., 2012).

### Comparison of Proposed Binding Mode of Ribbon and Globular α-Conotoxins

**FIGURE 7:**
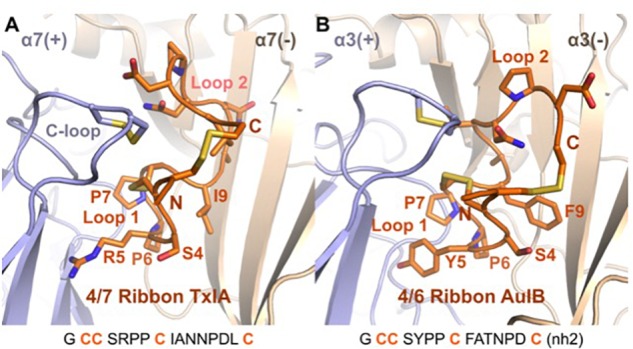
Comparison of the binding mode of ribbon TxIA **(A)** and ribbon AuIB **(B)** at the interface between two α7 or α3 subunits, respectively. The toxins are in orange, with their backbone in cartoon representation and their side chains in stick representation. The binding site principal (+) and complementary (–) subunits are shown in blue and wheat color, respectively. The vicinal disulfide bond of the C-loop is shown in stick representation. The two binding sites have been oriented similarly for convenient comparison. The sequence of the two toxins is shown at the bottom of the figure.

A binding mode was recently proposed for the ribbon α-conotoxin AuIB at the α3β4 nAChR (Wu et al., 2018), and this binding mode is similar to the MD-refined binding mode of ribbon TxIA, as illustrated in Figure 7. It was demonstrated that the sole binding site of ribbon AuIB is at the α3(+)α3(−) interface based on its subunit stoichiometry-dependent activity at α3β4 nAChR (Grishin et al., 2010). Ribbon AuIB was recently suggested to adopt a similar binding mode to that of the globular α-conotoxin on the basis of an Ala scan which revealed that most of the residues that are important for the activity of the globular isomer are also important for the ribbon isomer (Wu et al., 2018). Crystal structures of α-conotoxin/AChBP complexes showed that the first loops of the globular α-conotoxins overlap well, whereas the second loops adopt different conformations and interactions (Dutertre et al., 2007). Like the structures of globular α-conotoxins belonging to different loop-length subgroups bound to the AChBPs, the conformations of the Loop 1 of ribbon 4/7 TxIA and 4/6 AuIB overlay well in the binding sites but their second loop adopts different conformations. We have previously been able to explain the impact of more than 30 mutants of the complex between globular α-conotoxin ImI and the rat α7 nAChR, suggesting that our method can generate a reasonably accurate model of the receptor (Yu et al., 2011; Yu et al., 2012). The differences of binding modes observed after the two refinement methods seem therefore to originate from modeling the flexibility and interaction of the peptide. Interestingly, ribbon [P7A]AuIB has a structure similar to globular AuIB, but despite this change of conformation this mutation is innocuous (Wu et al., 2018), suggesting that ribbon AuIB would adopt a helical conformation by conformational selection. Similarly, ribbon TxIA also becomes more helical in both the MD- and ToxDock-refined models, suggesting that a helical conformation is optimal for interaction. The orientations of the loop 2 of ribbon TxIA and AuIB are different in the binding site, although position 9 (I9 of ribbon TxIA and Y9 of ribbon AuIB) was suggested to be buried at the interface with the complementary subunit for both peptides. Position 9 has been identified as a key determinant that modulates the activity of ribbon AuIB and it could also be involved in modulation of the activity of ribbon TxIA.

Because of their exceptional selectivity for certain ion channel or receptor subtypes, many conopeptides are used as tools in neuroscience and pharmacological research. Some of them have even been developed for clinical applications such as ω-conotoxin MVIIA (ziconitide), which is an FDA-approved analgesic for the treatment of intractable pain (Livett et al., 2004; Essack et al., 2012). In the case of α-conotoxins, the 4/7 α-conotoxins Vc1.1 and MII, the 4/3 α-conotoxin RgIA, and the 4/6 α-conotoxin AuIB, were found to have potent analgesic properties. Although there still exists some controversy regarding their physiological target (for a recent review see, Dutertre et al., 2017) these conotoxins are important lead structures. New methods for large-scale production of these conotoxins and development of a deeper understanding of their structure-activity relationships will accelerate their development into optimized tools and, hopefully, novel drugs.

## Author Contributions

AN: study design. YH, XW, RC, JG, RA, SD, QK, VH, and AN: experiments. YH, XW, RC, RA, DC, SD, QK, VH, and AN: data analysis. QK and AN: manuscript preparation. YH, DC, GK, and VH: manuscript editing. GK, VH, and AN: funding acquisition. All authors revised and approved the manuscript.

## Conflict of Interest Statement

The authors declare that the research was conducted in the absence of any commercial or financial relationships that could be construed as a potential conflict of interest.

## Supplementary Material

FIGURE S1 Expression **(A)**, purification **(B)**, and analysis of antagonistic potency **(C)** at α3β2 nAChR of the [R5N,I9H]TxIA double mutant.

FIGURE S2 Second step HILIC fractionation of purified expressed TxIA analogs and synthetic globular and ribbon TxIA isomers. Fractions containing the correct molecular mass are highlighted in color. The red colored fractions were generally used for all further experiments, orange colored fractions contained additional salt ions. The dashed line indicates the gradient of solvent B (90% ACN/0.043% TFA). MALDI MS traces indicate the monoisotopic molecular masses of the synthesized toxins. All reported masses are for [M + H]^+^ ions. Note that the synthetic globular TxIA was C-terminally amidated, explaining the difference in molecular mass to the synthetic ribbon and recombinant TxIA (Figure 2C).

FIGURE S3 Characterization of chemically synthesized ribbon TxIA. **(A)** UV absorbance chromatogram at 214 nm of synthetic TxIA ribbon. The minor peak is probably due to conformational exchange since the merging of the two peaks is observed at 55°C. ACN gradient from 0 to 100% in 2.5 min. **(B)** ESI-MS of synthethic TxIA ribbon. **(C)** Overlay of the amide region of the TOCSY spectra for recombinantly expressed TxIA (red) and synthetic ribbon TxIA (blue). Small differences in chemical shift for the H_N_ protons are observed for Cys3 and Cys16 between samples presumably due to a slight variation in pH between samples.

FIGURE S4 Comparison of the interactions between the position 5 of ribbon TxIA **(A)** and R5N **(B)** and R5D **(C)** variants at the α7 nAChR in the ToxDock-refined molecular models. The α7 nAChR is in blue and the toxins in orange. Hydrogen bonds are represented using dashed lines.

DATA SHEET S1 Atomic coordinates files in PDB format of the molecular models of the interaction between ribbon TxIA and the α7 nAChR. Two models are provided, one refined using a molecular dynamics simulation and the other refined using the ToxDock protocol.
