# Supplementary figures and images for: Periplasmic Expression of 4/7 α-Conotoxin TxIA Analogs in E. coli Favors Ribbon Isomer Formation – Suggestion of a Binding Mode at the α7 nAChR
Source: Front Pharmacol. 2019 May 31;10:577. doi: 10.3389/fphar.2019.00577 (PMC6554660; doi:10.3389/fphar.2019.00577)

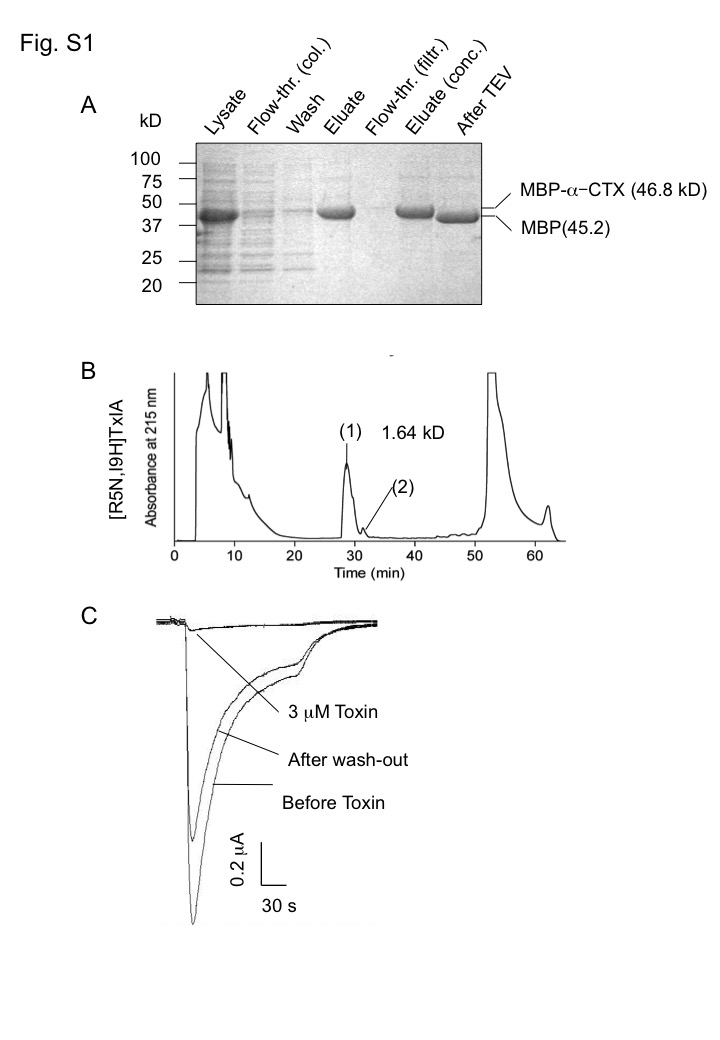

Supplement: FIGURE S1 — Expression (A), purification (B), and analysis of antagonistic potency (C) at α3β2 nAChR of the [R5N,I9H]TxIA double mutant. [file Image_1.JPEG]

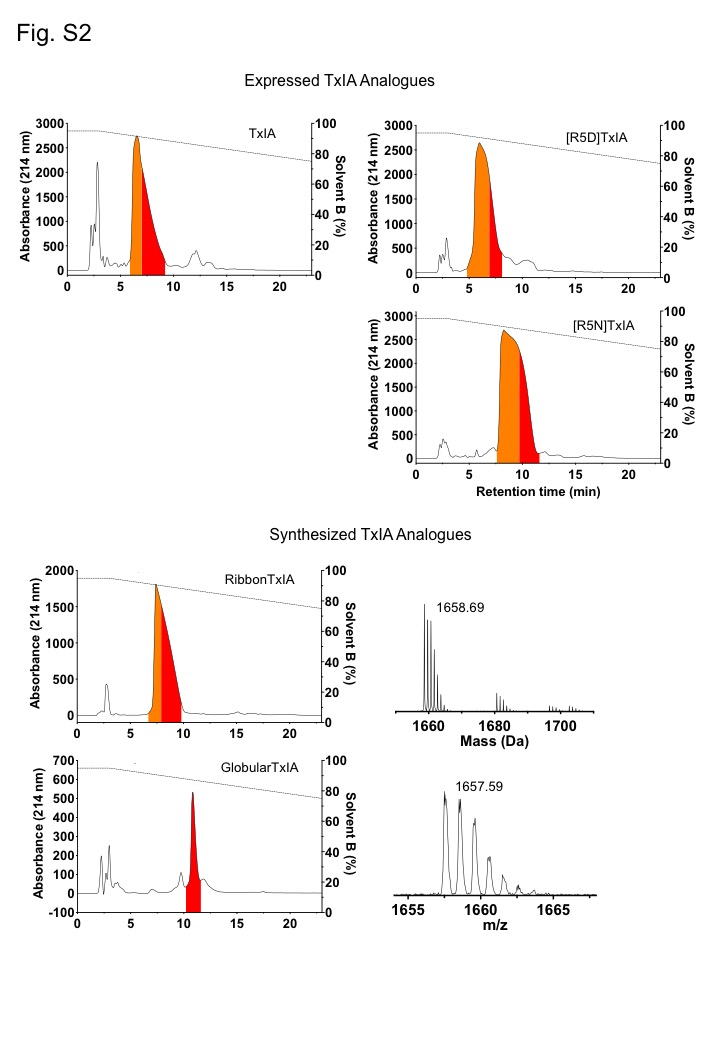

Supplement: FIGURE S2 — Second step HILIC fractionation of purified expressed TxIA analogs and synthetic globular and ribbon TxIA isomers. Fractions containing the correct molecular mass are highlighted in color. The red colored fractions were generally used for all further experiments, orange colored fractions contained additional salt ions. The dashed line indicates the gradient of solvent B (90% ACN/0.043% TFA). MALDI MS traces indicate the monoisotopic molecular masses of the synthesized toxins. All reported masses are for [M + H]+ ions. Note that the synthetic globular TxIA was C-terminally amidated, explaining the difference in molecular mass to the synthetic ribbon and recombinant TxIA (Figure 2C). [file Image_2.JPEG]

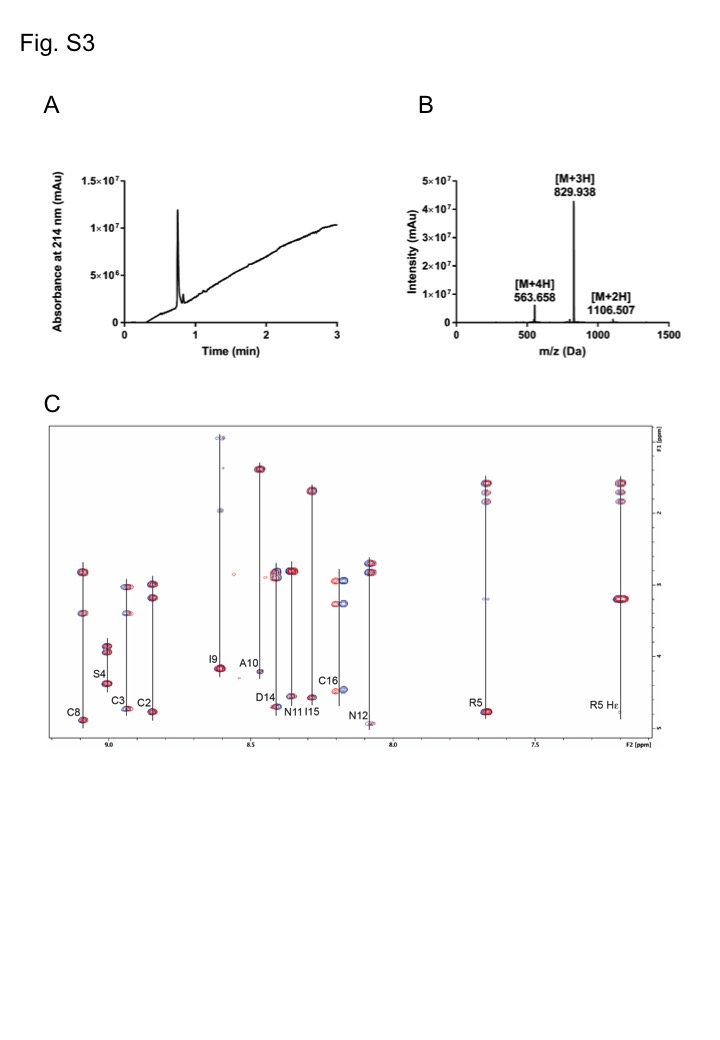

Supplement: FIGURE S3 — Characterization of chemically synthesized ribbon TxIA. (A) UV absorbance chromatogram at 214 nm of synthetic TxIA ribbon. The minor peak is probably due to conformational exchange since the merging of the two peaks is observed at 55°C. ACN gradient from 0 to 100% in 2.5 min. (B) ESI-MS of synthethic TxIA ribbon. (C) Overlay of the amide region of the TOCSY spectra for recombinantly expressed TxIA (red) and synthetic ribbon TxIA (blue). Small differences in chemical shift for the HN protons are observed for Cys3 and Cys16 between samples presumably due to a slight variation in pH between samples. [file Image_3.JPEG]

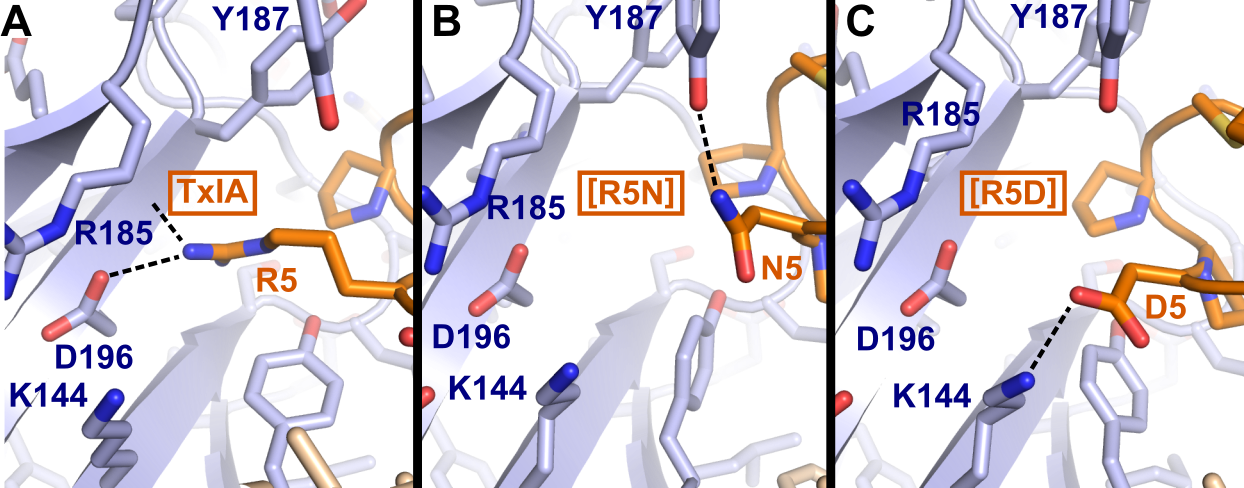

Supplement: FIGURE S4 — Comparison of the interactions between the position 5 of ribbon TxIA (A) and R5N (B) and R5D (C) variants at the α7 nAChR in the ToxDock-refined molecular models. The α7 nAChR is in blue and the toxins in orange. Hydrogen bonds are represented using dashed lines. [file Image_4.png]
